# A New Pronouncing Dictionary of Medicine

**Published:** 1892-09

**Authors:** 


A NEW PRONOUNCING DICTIONARY OF MEDICINE.—By John M. Keating, M. D., LL. D, and Henry Hamilton, with the collaboration of J. Chalmers DaCosta, M. D., and Frederick A. Packard, M. D. Published by W. B. Saunders, Philadelphia, 1892. Price: cloth, $5.00 net; sheep, $6.00 net.

THE publication of this work, since its announcement, has been awaited with no small degree of anxious expectation. It is a voluminous and exhaustive hand-book of medical and scientific terminology, with phonetic pronunciation, accentuation, etymology, etc., with an appendix containing important tables of bacilli, micrococci, leucomaines, ptomaines; drugs and materials used in antiseptic surgery; poisons and their antidotes; weights and measures; thermometric scales; new officinal and unofficinal drugs. So thorough has been the work of its authors, that it is but just to say that it reflects great credit both upon themselves and the publisher, and that it will be found a valuable and indispensable aid to the physician.

